# Near-Infrared Sensors for Onsite and Noninvasive Quantification of Macronutrients in Breast Milk

**DOI:** 10.3390/s22041311

**Published:** 2022-02-09

**Authors:** Candela Melendreras, Sergio Forcada, María Luisa Fernández-Sánchez, Belén Fernández-Colomer, José M. Costa-Fernández, Alberto López, Francisco Ferrero, Ana Soldado

**Affiliations:** 1 Department of Physical and Analytical Chemistry, University of Oviedo, 33006 Oviedo, Spain; uo257805@uniovi.es (C.M.); marisafs@uniovi.es (M.L.F.-S.); jcostafe@uniovi.es (J.M.C.-F.); 2 Department of Nutrition, Grasslands and Forages, Regional Institute for Research and Agro-Food Development (SERIDA), 33450 Villaviciosa, Spain; sforcada@serida.org; 3 Service of Neonatology, Department of Pediatrics, Hospital Universitario Central de Asturias, 33011 Oviedo, Spain; bcolomer@gmail.com; 4 Department of Electrical Engineering, University of Oviedo, 33204 Gijon, Spain; uo181549@uniovi.es

**Keywords:** breast milk quality control, chemometrics, handheld, spectroscopy

## Abstract

Breast milk is an optimal food that covers all the nutritional needs of the newborn. It is a dynamic fluid whose composition varies with lactation period. The neonatal units of hospitals have human milk banks, a service that analyzes, stores, and distributes donated human milk. This milk is used to feed premature infants (born before 32 weeks of gestation or weighing less than 1500 g) whose mothers, for some reason, cannot feed them with their own milk. Here, we aimed to develop near-infrared spectroscopy (NIRS) measures for the analysis of breast milk. For this purpose, we used a portable NIRS instrument scanning in the range of 1396–2396 nm to collect the spectra of milk samples. Then, different chemometrics were calculated to develop 18 calibration models with and without using derivatives and the standard normal variate. Once the calibration models were developed, the best treatments were selected according to the correlation coefficients (r^2^) and prediction errors (SECVs). The best results for the assayed macronutrients were obtained when no pre-treatment was applied to the NIR spectra of fat (r^2^ = 0.841, SECV = 0.51), raw protein (r^2^ = 0.512, SECV = 0.21), and carbohydrates (r^2^ = 0.741, SECV = 1.35). SNV plus the first derivative was applied to obtain satisfactory results for energy (r^2^ = 0.830, SECV = 9.60) quantification. The interpretation of the obtained results showed the richness of the NIRS spectra; moreover, the presence of specific bands for fat provided excellent statistics in quantitative models. These results demonstrated the ability of portable NIRS sensors in a methodology developed for the quality control of macronutrients in breast milk.

## 1. Introduction

Breast milk is a complex and highly variable fluid that provides nutrients and bioactive components for the correct growth and development of infants. The composition of breast milk changes throughout the lactation period, adapting to the nutritional needs of the rapidly growing newborn. Depending on the time of lactation, three types of milk can be distinguished: colostrum, transitional milk, and mature milk.

Breastfeeding is necessary for the development of newborns because it protects them from infections and diseases such as diabetes, obesity, and or hypercholesterolemia. Due to the multiple benefits of breast milk for infants, in the middle of the 20th century, milk banks were established. Currently, the World Health Organization and national and international pediatric associations consider breast milk banks as necessary to guarantee adequate nutrition for premature infants (born before 32 weeks of gestation or weighing less than 1500 g) or sick infants who, for whatever reason, cannot be fed with their mother’s milk.

Milk banks are a specialized service integrated into the neonatology units of hospitals. Their objectives are the promotion and support of breastfeeding; they are responsible for the selection of donors, and the storage, processing, analysis, and distribution of milk. To guarantee the safety of donated milk, it is subjected to a pasteurization process using the Holder method (62.5 °C for 30 min and then rapidly cooled in less than 15 min to 4 °C). In neonatal units, human breast milk contains 70 kcal/100 mL because it was obtained from women of term babies later in the lactation period. However, the amount of macronutrients in breast milk depends on different factors such as gestational age, feed, or sampling procedure [1] and no predictions can be established [2]. As such, when comparing preterm and term milk, the former one has less energy and protein than the latter. Considering these issues, one major concern when feeding neonates with breast milk from hospital banks is the nutritional adequacy of the milk in meeting the nutritional requirements of the neonate, because some nutrients such as protein are associated with neurodevelopmental outcomes [3].

To determine the quality of breast milk, nutritional analysis should be carried out. Nowadays, milk quality is controlled in laboratories using properly validated chemical reference methodologies such as Mojonnier, Folch, Gerber, or the Roese-Gottlieb method for fat quantification, and the Bradford method for protein elemental analysis. Another alternative used in some milk banks involves quantifying the referred compounds in milk analyzers using pre-calibrated medium or near-infrared (IR) instruments. To establish the quality of the results obtained by the IR technique, Fusch et al. compared eight different laboratory IR analyzers to quantify macronutrients in breast milk, concluding that research groups using these devices must be cautious about their measurements, finding that adequate sample preparation and instrumental calibration and validation are necessary following the Good Laboratory and Clinical Practices [4].

Given these considerations, the best method of guaranteeing the adequate nutrition of newborns is the use of onsite and real-time sensors that are able to quantify the macronutrients in breast milk. Moreover, due to the value and short supply of breast milk, a noninvasive analysis would be the best method to achieve final sample analysis. This type of analysis can be carried out just before consumption. Among the portable, noninvasive, and real-time analytical techniques for food analysis, near-infrared spectroscopy (NIRS) is a real-time, noncontaminating, and versatile technology capable of providing information on food quality attributes in situ. Moreover, no sample pretreatment or chemical reagents are required for the analysis, making it a waste-free technique, unlike traditional laboratory nutritional compositional analyses [5,6,7]. Nevertheless, there are some important limitations of NIRS analysis due to the strong absorption of water in the NIR region, which increases the background, preventing obtaining satisfactory results in quantitative analysis. Nowadays, the applications of NIRS have increased due to the development of aquaphotomics [8], a novel science and methodology that features water NIRS spectra in aqueous and biosystems for indirect analysis of components.

The drawbacks of using NIRS (Near Infrared Spectroscopy) with portable instruments are the sensitivity of the NIR signal, the narrow wavelength range of low-cost devices, and spectrum complexity. NIR spectra are difficult to interpret because the vibrations of different molecular bonds are involved in the same absorption band, resulting in overlapping information. However, to overcome these shortcomings and to extract information, it is necessary the use multivariate analysis [5,9,10]. By combining NIR spectra and appropriate multivariate analysis, it is possible to extract all the relevant information and to develop a fit calibration model that is able to quantify the macronutrients in breast milk both onsite and in real-time. This methodology will enable newborns to be fed with breast milk that is adequate for their development stage [11,12,13].

Qualitative models have been developed with portable instruments, which allow differentiation between colostrum, transition milk, and mature milk [14]; however, no information about breast milk quantification parameters or the effect of spectra chemometric pretreatments on the final calibration models with portable devices is available. NIRS methodologies were developed based on the use of handheld, portable NIRS, and were tested for the analysis of the three major components in cow raw milk (fat, protein, and nonfat solids) [15]. Because of this, the application of onsite NIRS technology to the study of breast milk can be an effective alternative for the characterization and control of the quality of donated breast milk received by milk banks. An important aspect to take into account, and studied by Kwan et al., is that quantitative NIRS studies on breast milk were carried out with high-performance laboratory equipment, that were acquired precalibrated, which can lead to errors due to a systematic displacement of the data or some inaccurate calibrations [16].

To the best of our knowledge, no studies have proved the effectiveness of low-cost, onsite, and easy-to-use handheld instruments in quantifying macronutrients in breast milk. The complexity of the analysis, the need for obtaining macronutrients quantification in real-time, and the small amount of available breast milk demand new methodologies able to meet all the above-detailed requirements.

As such, in this study, we constructed methodology based on the use of low-cost NIRS sensors and appropriate chemometric procedures for onsite, real-time quality control monitoring of breast milk. The aim was to provide the neonatology units and milk banks with a cheap and easy-to-use tool that is able to establish the quality of milk just before newborn consumption to enable real-time decision making and ensure adequate nutrient combinations for feeding newborns.

## 2. Materials and Methods

### 2.1. Milk Samples

**Figure 1:**
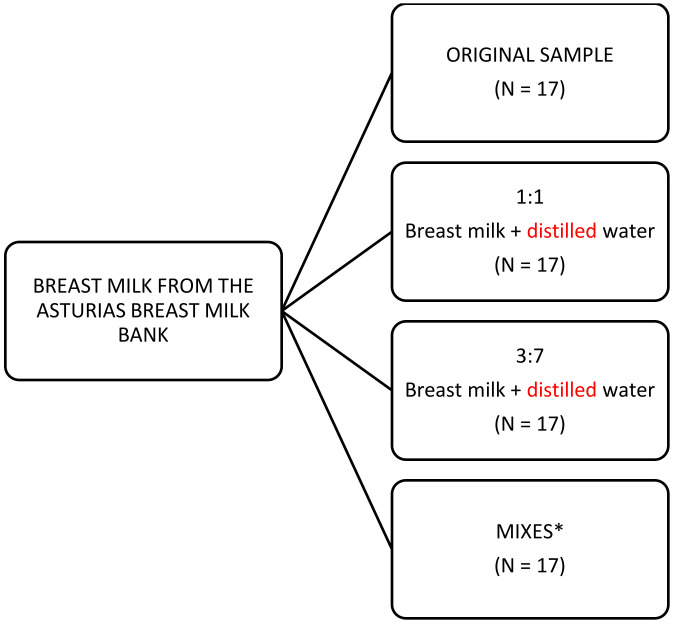
Scheme for battery sample preparation (N = 68). * Mixes (1:1) of following samples: 16 + 17, 10 + 4, 6 + 1, 7 + 1, 2 + 17, 3 + 16, 4 + 15, 5 + 1, 6 + 13, 7 + 12, 8 + 11, 9 + 10, 2 + 3, 5 + 8, 9 + 11, 12 + 13, and 3 + 15.

A total of 17 samples from the Asturias Breast Milk Bank (University Central Hospital of Asturias, HUCA, Oviedo, Spain) at different stages (colostrum, transitional milk, and mature milk) were used in this study. These samples covered the variability in breast milk for feeding newborns. However, for the development of an NIR calibration procedure, the recommended calibration samples minimum for any quality parameter is about 50 [16]. To increase the number of samples and the variability in the macronutrients content, we prepared three other batteries of 17 samples each (17 × 3) mixing different breast milk samples in a 1:1 proportion or diluting them 1:1 and 1:3.5 with distilled water (Figure 1). The final range of concentrations of all the parameters is shown in Table 1.

A total of 68 breast milk samples were involved in this NIRS research work. This initial set was separated into two groups: (i) a calibration set containing 53 samples because, when developing NIRS calibration models, a minimum of 50 samples is recommended [17]; (ii) an external validation set with 15 samples. Spectral data included in one or another set were randomly selected.

The statistics of the calibration and validation populations for all the parameters included for the quality control of breast milk are detailed in Table 1. The macronutrients analyzed were fat, crude protein (CP), real protein (RP), carbohydrates (CH), energy, and total solids (TS). As can be seen, we report two values for protein, crude protein, and true protein. The first is the protein content based on the total amount of nitrogen in breast milk; this value can include nonprotein nitrogen compounds, and true protein does not include these nonprotein nitrogen compounds [18]. All reference data were provided for the Asturias Breast Milk Bank.

All milk samples were stored frozen, allowed to thaw at room temperature before analysis, and then heated at 37 °C in a water bath. Once the samples were at temperature, they were homogenized by manual stirring and NIR analysis was carried out.

Each mother provided written informed consent for donating the samples for this study, which was approved by the institutional review board.

### 2.2. NIRS Spectra Collection

NIRS spectra were collected with a portable NIRS instrument (microPHAZIR Mod. 1624, Thermo Fisher Scientific Inc., Wilmington, MA, USA). This handheld instrument includes an electromechanical system (MEMS) and an incandescent tungsten light source for illumination, which is safe for users and ensures the integrity of the sample. It has a single, broad-spectrum InGaAs detector, which makes it a low-cost, energy-efficient device with a good spectral response. The scanning window or sampling area is 0.13 cm^2^, and the wavelength range of the device is 1596 to 2396 nm, with an approximate interval of 8.7 nm. It is compact and easy to handle, has a gun shape, and an integrated reference for easy calibration. For sampling, we used a liquid cup (Foss IH-0397, Foss NIRSystems, Silver Spring, USA), 45 mm high and 25 mm wide, with an optical path of 17 mm. This cuvette had a quartz wall (the wall of radiation incidence) and a rear wall of aluminum that reflected the NIR radiation and allowed the radiation to pass through the sample twice. Spectral information was collected in transreflectance mode, from direct exposure on the cuvette, and each NIRS spectrum is reported as the average of 5 scans. To obtain a representative spectrum of each breast milk, the collection procedure was as follows: each sample was divided in three aliquots and one spectrum was collected for each one. The final signature for each sample is reported as the averaged spectrum of the three subsamples. In the global procedure, a total of 204 scans (68 samples × 3 scans per sample) were collected as log 1/R, where R is reflectance, to build the spectra database of the 68 breast milk samples.

### 2.3. Data Processing

NIRS spectra collected with the handheld instruments are defined by 100 points in a range of wavelengths between 1596 and 2396 nm. Unscrambler X software (version 10.1, CAMO Software, Oslo, Norway) was used for chemometric developments. The calibration set was centered prior to performing the regression models by principal component analysis (PCA) to detect potential spectral outliers, and the regression procedure employed to build the calibration models using the global spectrum (all the wavelengths 1596–2396 nm) was partial least squares (PLS) [19]. The models were optimized using a random cross-validation method included in the Unscrambler X software package, with 20 segments and 3 samples per segment. The optimal number of PLS factors was established considering the minimum residual variance.

With the aim of minimizing the scattering phenomenon, the standard normal variate (SNV) mathematical pretreatment was applied to raw spectral data. After that, different derivative pretreatments were applied to the spectral data to minimize unforeseen variations and to improve calibration. The pretreatments code in this chemometric software can be summarized using a four-digit notation, where the first digit (a) refers to the order of magnitude of the Savitzky-Golay derivative (SG) (0 = underived spectrum, 1 = 1st derivative, 2 = 2nd derivative, etc.); the second digit (b) indicates the polynomial order of the derivate; and the third (c) and fourth (d) digits indicate the size of the left and right intervals, respectively, expressed in nanometers, used for the derivative smoothing calculation. A total of 18 different models (6 parameters × 3 chemometric strategies) were developed using different pretreatments of the breast milk samples and PLS as the regression model. According the four-digit notation, the chemometric strategies assayed in this research work were 0 0 0 0, 0 2 2 2, and 1 2 2 2.

The best-fitting equations were selected by the statistical criterion for each parameter, based on the lowest standard error of calibration (SEC) and standard error of cross-validation (SECV), the highest coefficient of determination for calibration, (R^2^), and coefficient of determination for cross-validation (r^2^) [20,21].

The external validation was evaluated based on the lowest standard error of prediction (SEP) and the best Student’s t-statistic for paired samples comparing the reference and NIRS method.

## 3. Results and Discussion

**Figure 2:**
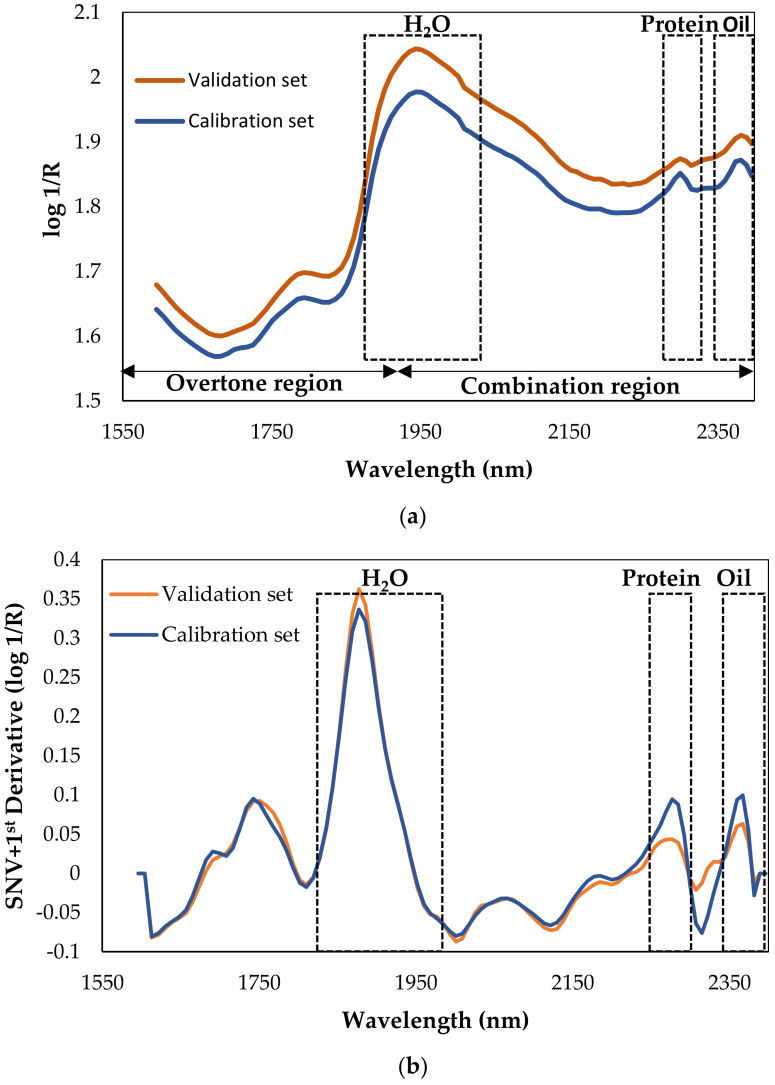
Average spectra of the calibration and validation sets: (**a**) raw spectra; (**b**) SNV + first derivative pretreatment.

In order to understand the information in the collected NIRS spectra, Figure 2 plots the raw and after-derivation values of the averaged spectra for the calibration and validation sets. We observed no differences between the populations, with the water band (O-H interactions) at 1950 nm being the most intense. At this wavelength, the band was a result of multiple overlapping bands, and it was directly influenced by chemical interactions with other molecular species in the sample [5]. Other important bands that could be associated with macronutrients in milk located at 2300 and 2380 nm. These are described as protein and fat (oil) bands, respectively [22]. Based on the aquaphotomic principle (strategy of monitoring a spectral band associated with a specific parameter, such as water, fat, protein, etc.), the location of these specifics bands (protein and fat) could help improve the calibration models for the aforementioned macronutrients.

**Figure 3:**
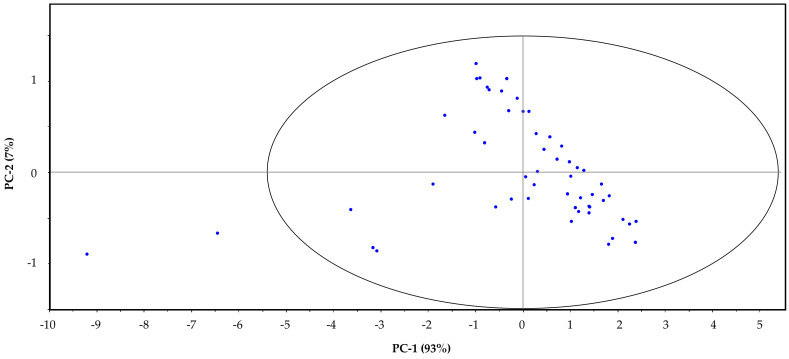
Principal component analysis with Hotelling’s T^2^ ellipse analysis for outlier detection in the calibration set.

After explaining the effect of vibrations associated with the macronutrients at different wavelength bands and their importance in the development of the NIRS procedure for the proposed parameters, we applied PCA with Hotelling’s T^2^ ellipse to raw NIR spectra of the calibration set to explore the spectra and detect outliers. As shown in Figure 3, the data outside the ellipse need to be checked because they are potential outliers. We can see that there are two samples located outside the ellipse: numbers 36 and 44. These samples were a mix of the originals 16 + 17 and 7 + 12, respectively. These results could be explained by operational error and not due to a compositional or spectral difference between these samples and all the others involved in this study. All original samples other than the mixed and diluted ones were satisfactory according PCA. Both outliers were deleted for the development of the final calibration models.

The next step was to run the calibration models with the global spectrum using PLS as a regression strategy and cross-validation with random groups. As detailed in the Section 2, different pretreatment procedures were evaluated to obtain the best calibration statistics, ranging from no pretreatment to scattering correction (SNV) plus first derivative. As shown in Table 2, a total of 18 calibration models applying different pretreatments, both with and without scattering correction and derivatives, were used to quantify six parameters (fat, crude protein, raw protein, carbohydrates, energy, and total solids) for quality control of breast milk.

As shown in Table 2, for all parameters, with the exception of raw protein (R^2^ = 0.58), the R^2^ calibration coefficient obtained was higher than 0.7, and the best performance was exhibited by fat, energy, and total solids with R^2^ values all higher than 0.9. These results for fat could be explained due to spectrum bands: as shown in Figure 2, a specific band for fat is noted around 2380 nm, as was observed for the breast milk samples. The presence of this band allowed us to obtain excellent calibration results with raw spectra data, without any pretreatment (R^2^ = 0.91). Notably, fat is one of the most important parameters when characterizing donated breast milk as it is the main source of energy for newborns. Furthermore, it provides essential nutrients such as fat-soluble vitamins and polyunsaturated fatty acids [23]. These results showed that the NIR spectra successfully captured quantitative variations in fat, showing the richness of the NIRS spectra.

Similarly, quantitative results could be obtained using the proposed procedure, because NIRS regression coefficients were obtained for energy and total solids, with R^2^ values higher than 0.9 [5]. Precision values calculated as coefficient of variation (CV% = 100 × SEC/mean calibration set reference values) ranged between 10% and 15% for all the parameters involved in this study. Similar results were obtained in previous research [16] when comparing the results produced by different devices and reference methods. For these parameters, the best results were obtained after applying SNV and the first derivative to the raw spectra. For RP, CP, and CH, not many differences were observed when developing PLS regression with or without pretreated spectra. The R^2^ value for protein data was higher than 0.75 and for CH, it was higher than 0.8. These values indicated that all the developed models can be used for quantitative analysis.

After studying the calibration statistics of the NIRS multivariate models in depth, the next step was to select the best chemometric models to quantify each macronutrient in breast milk. This choice was made based on the criterion detailed in the Section 2 (the highest r^2^ value and the lowest SECV) and on the comparison between SEC and SECV, because a gap between SEC and SECV is related to large differences between calibration and prediction results, which indicate that the calibration model was not robust.

Although few differences were found when comparing the NIRS calibration and cross-validation statistics, the best results for fat (r^2^ = 0.841, SECV = 0.51), proteins (RP, r^2^ = 0.512, SECV = 0.21), and carbohydrates (r^2^ = 0.741, SECV = 1.35) were obtained when no pretreatment was applied to the NIR spectra. For energy (r^2^ = 0.830, SECV = 9.60), SNV plus the first derivative obtained satisfactory results. As shown in Table 2, the best R^2^ value for TS was obtained with a first-derivative pretreatment; however, the difference observed between cross-validation and calibration data was too large, indicating that the results were not robust and the validity of that model was limited. For these reasons, we determined that the best model for TS quantification was that without applying any pretreatment to the spectra (r^2^ = 0.685, SECV = 2.42).

The energy and TS parameters showed the largest differences in SECV and SEC values. The reason for these results may be that both parameters are indirect. This means that they are not directly related to a molecular bond. However, the robustness of the calibrations could be improved by enlarging the sample size and the variability in the breast milk. An update of the calibration models is required when new samples are considered. However, no conclusions could be reached with this calibration and cross-validation data, as an adequate statistic test is needed to quantify the SEP (external validation).

After selecting the best treatments, to confirm the validity of the developed models, we then externally validated the models, predicting all the macronutrients by using the selected models. As detailed in the Section 2, 15 breast milk samples with reference data were selected for external validation and all the parameters were quantified. After quantifying all the breast milk parameters with the developed models, Student’s *t*-test was applied to compare the results obtained when analyzing samples using the reference and onsite NIRS method.

As detailed above, the final acceptance or evaluation of the NIRS calibration models necessitated an external validation including samples not involved in the calibration procedure. In this work, 15 breast milk samples were included in the external validation set and quantified with the selected calibration models developed in this study. The selected methods and external validation statistics are detailed in Table 3.

As shown in Table 3, we compared the SEP and SECV values for the selected models. The ratio between SECV and SEP ranged between 0.817 and 1.035 for all parameters except for TS (0.533). The similarity between SECV and SEP confirmed that no difference was found between the external and cross-validation predicted errors, indicating a sufficiently robust calibration for all parameters [24]. For TS quantification, an improvement in the models is required by including new samples and enhancing the multivariate model.

Another statistic that can explain model reliability in NIR spectroscopy is RPD (RPD = standard deviation/SEP). For this parameter, three categories can be defined: (1) excellent models, with RPD > 2; (2) fair models, with 1.4 < RPD < 2; and, (3) unreliable models, with RPD < 1.4 [25]. These values have been applied in NIRS studies; however, no statistical basis has been used to establish these thresholds. Moreover, researchers developed useful calibration models with RPD values lower than the proposed standard values [25]. Considering this statistic in Table 3, we the fat and energy models are categorized as excellent and the RP and HC models as fair. For TS and CP, the range of and variability in the samples should be increased to improve the NIR statistics and obtain a valuable model.

After determining the fit and quality of the calibration models, and taking into account the coefficients of determination and calibration or cross-validation errors, a statistical test including SEPs and reference data was conducted to evaluate the prediction errors of the multivariate NIRS models. In this work, we selected Student’s *t*-test to compare paired samples. We applied a paired difference *t*-test to compare the results obtained when samples were analyzed by the reference and NIRS methods. Then, we used the set of differences to build the t-statistic using the mean and standard deviation of the differences. Student’s *t*-test is a useful statistic strategy for comparing two data sets of quantitative results obtained with different analytical methods. In this study, we compared the reference and predicted data of all samples included in the validation set (N = 15). The results showed that there were no differences when considering a confidence level of 95% because, for all parameters, the calculated *t*_student_ was is lower than 2.145 (theoretical value of t for 14 freedom degrees and 95% confidence level). Another parameter that we used to characterize the proposed methodology was accuracy. We calculated the accuracy for all the macronutrients involved in this study, and the best results were obtained for fat and energy with a value of 94%.

We could find no information about analytical methodologies for onsite and real-time quantification of the macronutrients in breast milk. Table 4 summarizes the most relevant studies conducted using NIRS technology, including the instrumentation employed to quantify macronutrients (high-performance laboratory instruments). Only dos Santos et al. [14] used a portable NIRS instrument to classify breast milk in colostrum, transition milk, and mature milk, which are the three stages of the lactation period.

Focusing on spectroscopic laboratory methodologies, previous researchers evaluated and compared the use of near- and mid-infrared instruments vs. reference methodologies using high-performance laboratory devices [28]. More specifically, the macronutrients quantified were fat, protein, and carbohydrates. The NIRS instruments employed were precalibrated NIRS laboratory devices, scanning in a range of 1200–2400 nm. No information about the statistics of calibration models were included in this work; however, external validation statistics can be compared with our results. The values of coefficients of determination detailed by Fusch et al. for validation were 0.76 for protein, 0.01 for lactose, and 0.79 for fat. In our study, employing a portable device and developing our own calibration models, the coefficient of determination of cross-validation was 0.52 for protein, 0.74 for HC, and 0.84 for fat MISSING [28].

Table 5 compares the external validation statistics obtained with laboratory instrument vs. the developed portable-device methodology by computing the linear regression of the reference and predicted NIRS data [28]. To evaluate the random error in the prediction based on regression results, the S_y/x_ statistic (random error in the y-direction, y-direction indicates the prediction values and x-direction reference values) was calculated. Comparing these data with SEP values (Table 3) for fat, RP, and HC, we observed that the regression error (S_y/x_) was equal or lower than the error obtained in external validation (SEP), confirming the validity of the developed models.

Another NIRS strategy for macronutrients milk analysis was carried out by de la Roza et al. [2], who developed in-house NIR calibrations with a high-performance laboratory instrument (working range 400–2500 nm) for fat, protein, and nonfat total solids in cow milk. The best result, with an R^2^ value of 0.971, was obtained for the quantification of fat, applying mathematical SNV, detrend, and second derivative pretreatments. The R^2^ value is similar to that obtained in this work (0.910). However, for total solids, the values of the calibration coefficient of determination for cross-validation were lower than 0.700 (r^2^ = 0.612) [2], and better results, above 0.750, were obtained using our handheld portable instrument (r^2^ = 0.787).

## 4. Conclusions

In this work we focused on the development of a real-time and simple methodology to quantify the macronutrients in breast milk. Notably, the implementation of this procedure requires the use of low-cost and handheld NIRS instruments. Moreover, expert personnel are not required for analyzing samples, facilitating the quality control procedure in the feeding of newborns in neonatology units.

In this paper, we demonstrated the feasibility of using a cheap and easy-to-use handheld NIRS instrument with a narrow scanning range, from 1596 to 2396 nm, and a small scanning window to control the quality of breast milk. By using a multivariate strategy and different pretreatments, we developed quantitative calibration models to determine the energy, fat, carbohydrate, and protein contents in this type of sample, with coefficients of determination for calibration (R^2^) higher than 0.79 for all parameters. These results, obtained with a limited number of breast milk samples (68 samples), can be considered as a first step in the development of an appropriate method, even if more work needs to be carried out to improve the equations and to include more samples in the database. The interpretation of the results obtained for fat analysis demonstrated the richness of NIRS spectra because the presence of specific bands shows excellent potential for constructing useful quantitative models.

Prior to implementation, external validation was required. We tested the proposed methodology with breast milk samples not involved in the calibration procedure, and nonsignificant differences were observed when comparing the reference and portable NIRS methods. The accuracy obtained for the tested parameters was higher than 90%. Our results suggest that NIR sensor measurements of macronutrients are acceptable for clinical use in breast milk banks. The limitation of the proposed method is related to the range of values for each nutrient; however, further improvements can be achieved by including new samples to update this first calibration method.

## Figures and Tables

**Table 1:** Statistics of macronutrients and energy in breast milk (N = 68).

Calibration Set (N = 53)					Validation Set (N = 15)			
	Mean	Max	Min	SD	Mean	Max	Min	SD
Fat ^1^	2.39	5.30	0.51	1.25	2.60	4.60	0.57	1.58
CP ^1^	0.87	2.50	0.27	0.50	0.78	1.70	0.33	0.39
RP ^1^	0.72	2.00	0.21	0.40	0.69	1.40	0.27	0.34
CH ^1^	5.80	8.80	2.34	2.56	5.58	8.40	2.31	2.63
Energy ^2^	49.30	86.00	15.60	22.06	50.09	81.00	15.90	26.36
TS ^1^	7.42	14.60	3.27	4.02	8.04	14.50	3.27	4.17

Max: maximum, Min: minimum, SD: standard deviation, RP: raw protein, CP: crude protein, CH: carbohydrates; TS: total solids. ^1^ g/100 mL; ^2^ Kcal/100 mL.

**Table 2:** Calibration and cross-validation statistics for breast milk samples using partial least squares regression.

Math Pre-Treatment	Parameter	R^2^	SEC	r^2^	SECV
0 0 0 0	Fat	0.910	0.37	0.841	0.51
	CP	0.782	0.19	0.508	0.30
	RP	0.797	0.14	0.512	0.21
	HC	0.874	0.91	0.741	1.35
	Energy	0.922	6.17	0.791	10.39
	TS	0.787	1.86	0.686	2.42
SNV 0 2 2 2	Fat	0.876	0.44	0.795	0.58
	CP	0.725	0.25	0.498	0.35
	RP	0.580	0.22	0.411	0.27
	HC	0.860	0.94	0.593	1.65
	Energy	0.835	8.96	0.756	11.32
	TS	0.709	2.13	0.529	2.77
SNV 1 2 2 2	Fat	0.826	0.52	0.779	0.61
	CP	0.796	0.22	0.524	0.36
	RP	0.787	0.16	0.482	0.25
	HC	0.894	0.83	0.699	1.42
	Energy	0.927	5.94	0.830	9.60
	TS	0.929	1.07	0.685	2.20

SNV: standard normal variate, N1N2N3N4: Savitzky-Golay derivative order, polynomial order of derivative, left, and right intervals for the derivative smoothing; R^2^: coefficient of determination for calibration, SEC: standard error of calibration, r^2^: coefficient of determination for cross-validation, SECV: standard error of cross-validation, RP: raw protein, CP: crude protein, CH: carbohydrates, TS: total solids.

**Table 3:** External validation statistics used for predicting nutritive parameters of breast milk (validation set, N = 15).

	Math Pre-Treatment	SECV	SEP	SECV/SEP	RPD	Accuracy %	*t*_student_ Reference vs. Predicted
Fat	0 0 0 0	0.510	0.579	0.881	2.7	94	1.21
CP	SNV 1.2.2.2	0.359	0.426	0.843	0.9	114	0.57
RP	0 0 0 0	0.210	0.203	1.035	1.7	92	0.69
HC	0 0 0 0	1.347	1.630	0.826	1.6	108	1.36
Energy	SNV 1.2.2.2	9.603	11.757	0.817	2.2	94	1.74
TS	0 0 0 0	2.420	4.541	0.533	0.9	115	1.57

SNV: standard normal variate, N1N2N3N4: Savitzky-Golay derivative order, polynomial order of derivative, and left and right intervals for the derivative smoothing, SECV: standard error of cross-validation, SEP: standard error of prediction, RPD = standard deviation of the validation set/SEP; Accuracy %: 100 − ((reference value − predicted value)/reference value) × 100), *t*-critical value for 95% confidence and 14 degrees of freedom = 2.145, RP: raw protein, CP: crude protein, CH: carbohydrates, TS: total solids.

**Table 4:** An overview on reported NIRS-based methods for breast milk analysis.

Reference	Device	Lab/Portable	Wavelength Range λ (nm)	Sample Size (N)	Analyzed Parameters
Corvaglia [26]	Fenir 8820, Esetek	Lab	700–2750	124	Fat and nitrogen contents
Sauer [27]	SpectraStar 2400 Near Infrared Analyzer, Unity Scientific	Lab	1200–2400	52	Fat, protein, and carbohydrates
Fusch [28]	SpectraStar 2400 Near Infrared Analyzer, Unity Scientific	Lab	1200–2400	1188	Fat, protein, and carbohydrates
dos Santos [14]	MicroNIR™ 1700, JDS Uniphase Corporation	Portable	910–1676	198	Qualitative (colostrum, transition milk, and mature milk)
Present study	MicroPHAZIR Mod. 1624, Thermo Fisher Scientific Inc.	Portable	1396–2396	68	Fat, crude protein, raw protein, carbohydrates, total solids, and energy

Lab: laboratory instrument; N = number of samples involved in the study.

**Table 5:** Comparison of external validation statistics: Ref. [28] (laboratory instrument) vs. the proposed methodology (portable device).

	Portable	Laboratory	Portable		Laboratory
	Linear Regression		r^2^	S_y/x_	r^2^
Fat	y = 0.69x + 0.72	y = 0.55x + 1.25	0.85	0.547	0.79
RP	y = 0.77x + 0.16	y = 0.55x + 0.54	0.67	0.154	0.76
HC	y = 0.95x + 0.60	y = 0.02x + 5.69	0.01	0.904	0.89

RP: raw protein, CH: carbohydrates, r^2^: coefficient of determination, S_y/x_: random errors in the y-direction, y: prediction values, x: reference values.

## Data Availability

Data sharing not applicable.
